# Synthesis of a Hybrid Membrane of Polysulfone with Zinc Oxide for Cleaning Textile Effluents

**DOI:** 10.1021/acsomega.4c04698

**Published:** 2024-08-15

**Authors:** Suzanna Rani Cristina Alves de Sousa, Késia Karina de Oliveira Souto Silva, Ivan Alves de Souza, Amanda Melissa Damião Leite

**Affiliations:** † Postgraduate Program in Textile Engineering (PpgET), Federal University of Rio Grande do Norte (UFRN), Campus, Natal, Rio Grande do Norte 59078-900, Brazil; ‡ Plasma Materials Processing Laboratory (LabPlasma), Federal University of Rio Grande do Norte (UFRN), Campus, Natal, Rio Grande do Norte 59078-900, Brazil

## Abstract

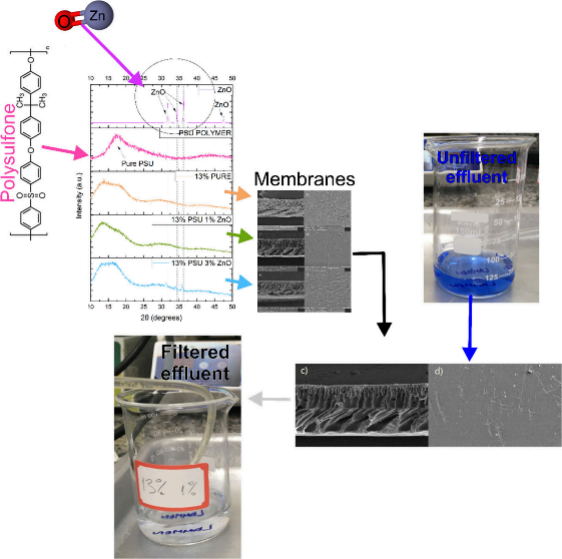

This study aimed to investigate the influence of the ZnO concentration on the structure of a membrane for effluent filtration, varying this concentration from 0% to 3%. To analyze the results, X-ray diffraction tests, Fourier-transform infrared spectroscopy, apparent porosity, atomic force microscopy, and scanning electron microscopy were used, all of which were employed for the characterization of the produced membranes. The solution simulating the effluent was analyzed before and after the filtration process to assess the filtration results. The conducted tests reported results for filtered solution flow, turbidity, pH, dissolved oxygen, and electrical conductivity. All these results indicated that the membrane with the best performance in terms of cleanliness and the amount of filtered effluent was the one produced with a 13% hybrid polysulfone loaded with 1% ZnO in its structure.

## 1 Introduction

Wastewater treatment in the textile industry is a field that has grown significantly in recent years. Large-scale production requires sustainable alternatives in all procedures used in the textile industry to minimize negative environmental consequences, with an emphasis on fiber processing and textile material development processes.^1−3^ These treatments are often based on biological methods^4^ and physicochemical methods.^5^

Biological methods involve exposing microorganisms such as enzymes, fungi, yeast, algae, and bacteria to pollutants so that they can be effectively cleaned by eliminating undesirable residues.^6^ As for physicochemical methods, they consist of various techniques and processes such as photodegradation,^7^ electrochemical processes,^8^ coagulation–flocculation,^9^ and membranes,^10^ among other processes that are also effective in removing pollutants in liquid media.

However, carrying out these processes requires specific temperature and pH conditions for proper operation, care for maintaining living organisms, and the possibility of easy enzyme inactivation, which could impair the treatment.^4^ Another drawback of these processes is that the inputs often can only be used once and discarded after treatment, besides not providing the possibility of reusing the waste from textile material processing.^11^

One of the techniques that stands out is the membrane separation process,^12^ which consists of filtering water for effluent treatment using polymeric membranes,^13−15^ where several polymers have been used for this purpose.^16−18^ This technique has two distinct advantages over others: membranes can be reused multiple times, and it also allows for selective separation of pollutants, enabling the recovery of substances that can be used again due to their ability to separate permeates and concentrates.^19^ Even so, some polymers, despite showing good filtration results, do not fully reach their potential due to the inherent hydrophobicity of these materials. To address this issue, some authors are using zinc oxide (ZnO) mixed into the membranes because, in addition to its antibacterial, antifungal, and anticorrosive properties,^20−22^ it can make the membrane more hydrophilic.^23^

In light of this, this work consists of using polysulfone (PSU), one of the polymers widely used by several researchers due to its good properties and easy handling. Its use is due to its high physical and thermal resistance, as well as excellent chemical stability, making it ideal for applications in adverse conditions such as those found in industrial effluent filtration,^24−27^ and together with zinc oxide, it has shown good results in degrading pollutants and dyes in water before disposal into the environment.^2^ The aim is to produce a membrane with high efficacy in cleaning effluents as well as to assess the relationship between the percentage of ZnO and the level and quality of filtration for effluent cleaning after treatment with the produced membrane.

## 2 Experimental Procedure

### 2.1 Materials

The methodology used in this study consisted of producing membranes of Udel P-3500 polysulfone, supplied by Solvay, with the addition of zinc oxide (ZnO) at 1% and 3% loading. The membranes were manufactured by using 1-methyl-2-pyrrolidone (NMP) as a solvent. They followed the following steps: preparation of pure polymer–solvent and hybrid solutions, stirring for 24 h, deposition of the solution on glass plates, immersion in distilled water for film formation, and drying for 24 h.

### 2.2 Procedures

Membranes were produced with 13% polysulfone in 87% NMP and hybrid membranes with 1% and 3% ZnO loads, based on the weight of the polymer. The membranes were subjected to flow tests in the Millipore Amicon 200 mL filtration cell with an effective area of approximately 0.00283 m^2^, capable of withstanding up to 75 psi. Flow tests were conducted with simulated effluent at 30 psi and 25 °C.

### 2.3 Characterizations

Additionally, the membranes were characterized using X-ray diffraction (XRD), fourier-transform infrared (FTIR) spectroscopy, contact angle measurement, apparent porosity, atomic force microscopy (AFM), and scanning electron microscopy (SEM). Pure zinc oxide, PSU polymer, and membranes were analyzed by XRD. FTIR spectroscopy analyzed the membranes to obtain information about their chemical composition. The contact angle was measured with distilled water at room temperature.

Immersing membrane samples in distilled water determined the apparent porosity, and we weighed them before and after the process. Atomic force microscopy (AFM) was used to analyze the surface topography of the membranes and identify roughness parameters. Scanning electron microscopy (SEM) allowed for the analysis of the membrane structure, including its surface and cross-section.

For the production of simulated textile effluent, a reactive textile dye, BLUE BG-R, was used at a concentration of 20 ppm. The characteristics of the simulated effluent were analyzed for turbidity, pH, dissolved oxygen, and electrical conductivity. The effluent color was measured with a UV–vis spectrophotometer, turbidity with an Instrutherm-TD-300 turbidimeter, pH with KASVI K36-014 model strips, temperature with an analogue thermometer, and dissolved oxygen with a MO-900 oximeter. Electrical conductivity was measured with a Water Lucity Meter.

## 3 Results and Discussion

### 3.1 FTIR Analysis

**Figure 1:**
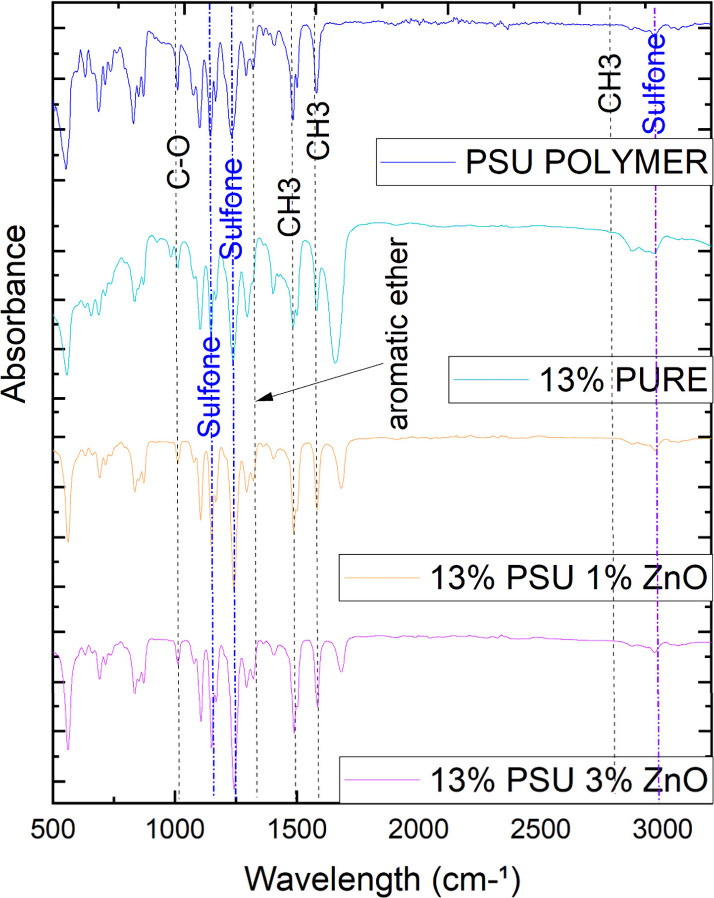
FTIR analysis of hybrid membranes, pure polysulfone membrane, polysulfone polymer, and zinc oxide.

The FTIR analyses shown in Figure 1 indicated that adding ZnO load to PSU membranes did not cause significant changes when compared to the membrane without ZnO addition and those with 1% and 3% additions. This is evident as the peaks corresponding to the polymer (peaks at 1149 cm^–1^, 1240 cm^–1^, and 2970 cm^–1^) appear in all analyses with minor changes in intensity, thereby not compromising the excellent properties of the polymer. Such a result also ensures that the alteration in the polymer composition at the used proportions may yield filtration results similar to those achieved by authors who solely used PSU in the production of their membranes.^28−31^

### 3.2 XRD Analysis

**Figure 2:**
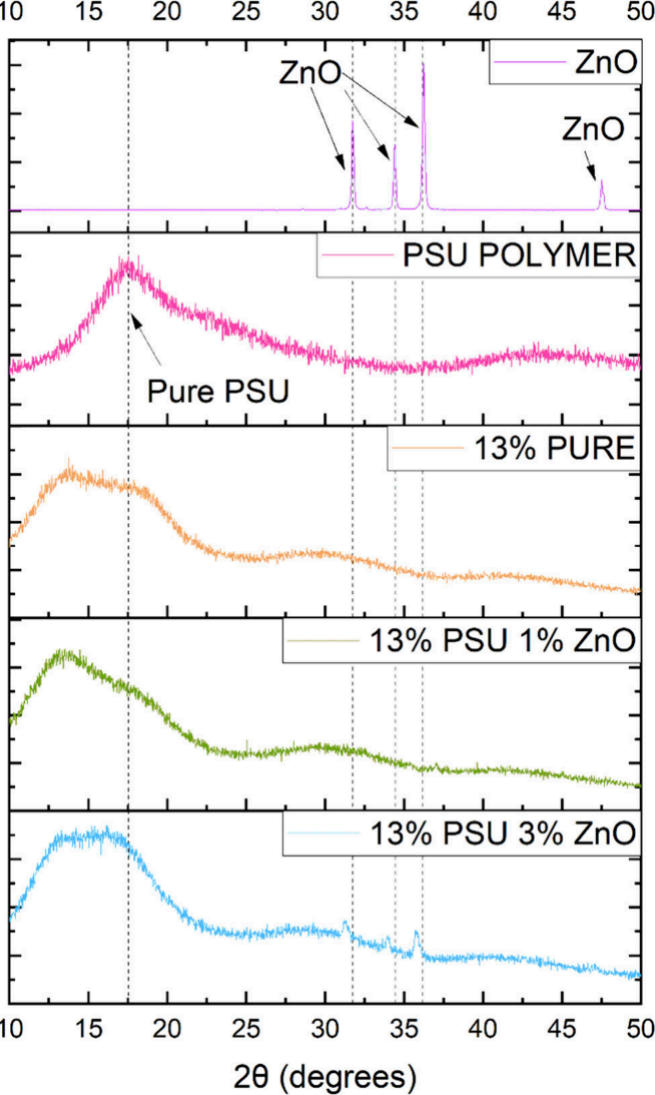
Comparison of XRD analyses of hybrid membranes, pure polysulfone membranes, polysulfone polymer, and zinc oxide.

Corroborating with the FTIR analyses, the XRD results in Figure 2 also showed that adding ZnO particles did not cause significant changes in the loaded membranes. However, this time it is possible to see that the membrane with 3% ZnO exhibits three out of the four main characteristic peaks of zinc oxide.^32,33^ The fourth peak is likely masked by the high intensities of the peaks related to PSU, making it so that even at a higher concentration the less intense peak of zinc oxide cannot be revealed by the analysis in question. However, it is already an indication that its presence in the membrane structure at this proportion may cause some alteration in the behavior of the membranes with 3% ZnO. This is confirmed by checking the contact angle analyses.

### 3.3 Contact Angle Analysis

**Figure 3:**
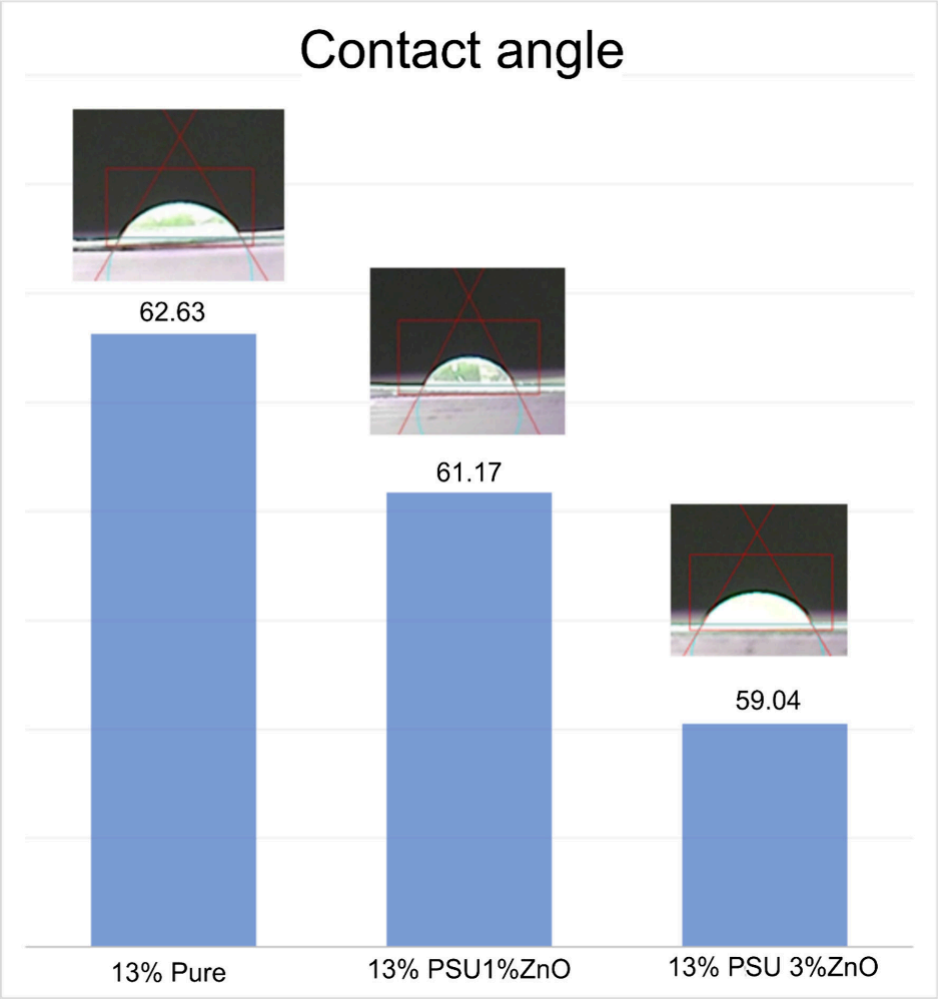
Average values obtained in the contact angle analysis of pure and hybrid membranes.

This analysis reveals that the addition of ZnO (which is a hydrophilic material) to the membrane composition has a beneficial effect on the material’s wettability,^34^ As can be seen in Figure 3, the sample with the highest contact angle and, therefore, the lowest wettability was the sample without the addition of zinc oxide. On the other hand, the sample with 1% showed a slight improvement in its wettability because the contact angle had a slight decrease. The sample with the best wettability had the highest percentage of ZnO, showing a reduction in the contact angle of approximately 6% compared with the pure sample. Although this result was somewhat insignificant, this behavior is directly related to the subsequent results and, consequently, to the filtration results, as we will see later on.

### 3.4 Apparent Porosity Analysis, SEM, and AFM

The filtration medium must have high porosity to achieve high flow but a controlled pore size to attain high selectivity. Porosity is crucial in effluent filtration, because it allows the filtering medium to retain unwanted particles and substances present in the effluent while permitting the passage of the solvent or clean liquid. To achieve such a result, it was necessary to use eq 1, where *Pm* and *Ps* are the weights of the wet and dry samples, respectively.
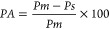
1The higher the porosity of the membrane or filter, the greater the capacity to retain particles of different sizes.^35−37^

**Figure 4:**
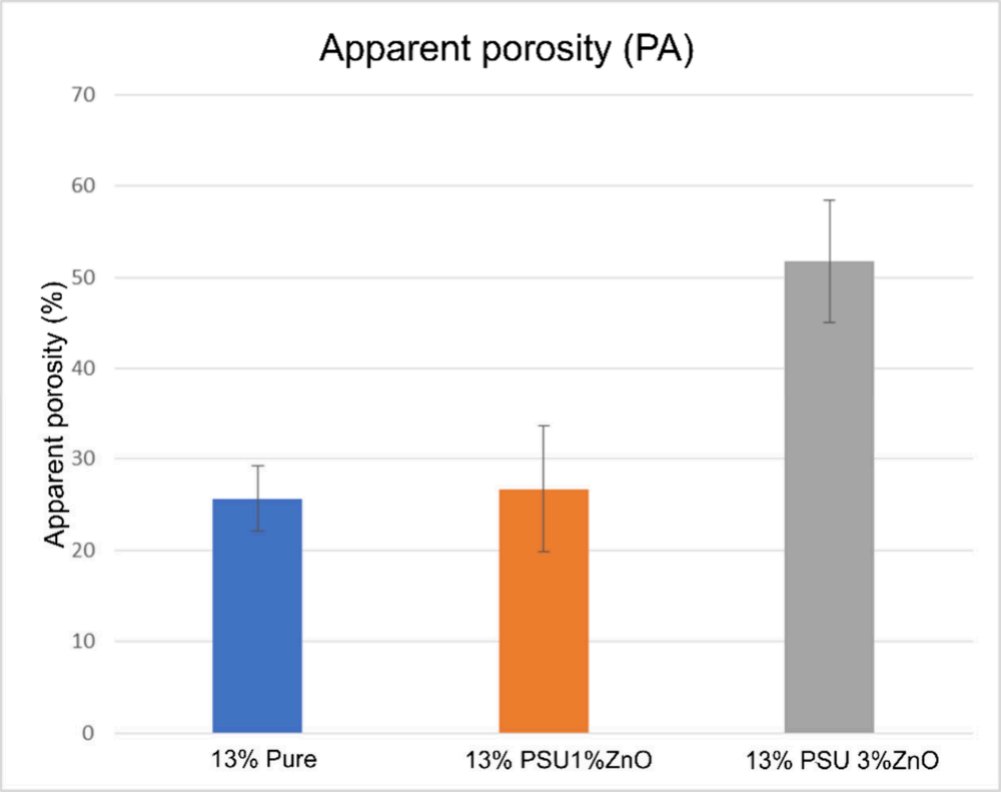
Apparent porosities of the membranes.

**Figure 5:**
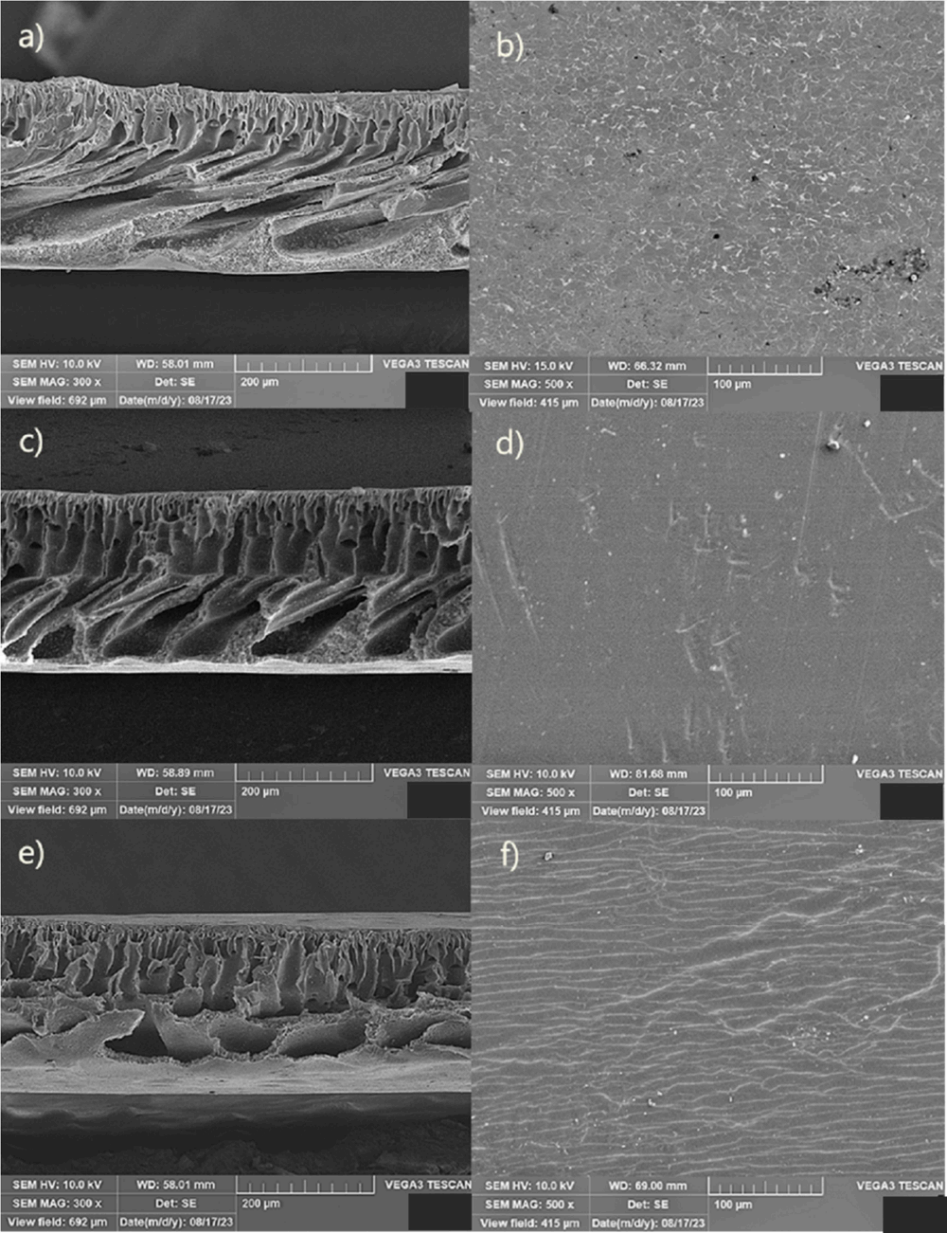
SEM cross-sectional and top images of pure PSU membrane (a, b), PSU + 1% Zno (c, d), and PSU + 3% ZnO (e, f).

The result of the apparent porosity shown in Figure 4 had virtually the opposite wettability behavior, meaning that higher porosity leads to better material wettability. However, when we analyze the SEM images of the samples in Figure 5b,d,f, we see that the same is not valid. The samples without ZnO and with the highest percentage of zinc oxide have a rougher appearance than those with 1%. This is explained by analyzing the cross sections of the samples in Figure 5a,c,e. It can be seen in these images that the upper structure of the samples indeed has differences.

We can see that the samples have significant differences on the surface, which explain the difference in the surface roughness. However, when analyzing the internal structure in Figure 5a,c for PURE PSU and PSU + 1% ZnO, respectively, both have similar internal profiles, with grooves connecting the upper surface of the membrane to the lower surface, differing only in the lower surface of the membrane. In the pure membrane, the thickness of the lower layer is greater than that of the sample PSU + 1% ZnO. On the other hand, the sample PSU + 3% ZnO has an entirely different internal structure from the previous two. However, upon reanalyzing Figure 5b,f, it is possible to notice that both samples have higher roughness than the sample with 1% ZnO (Figure 5d). To confirm this, it is sufficient to analyze the results of the AFM analyses.

**Figure 6:**
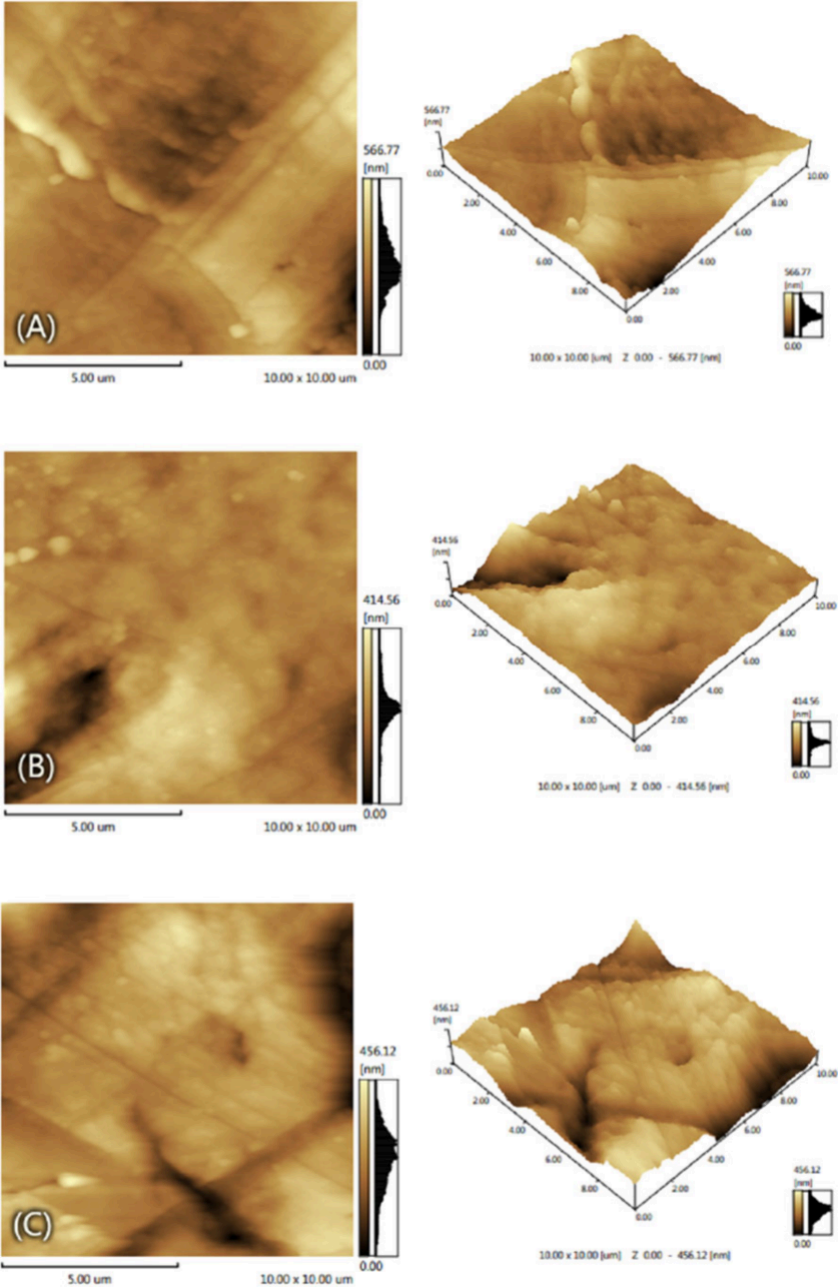
AFM images of pure membrane (A), with 1% (B) and PSU + 3% ZnO (C).

The AFM analyses (Figure 6) of the samples indeed confirm the higher roughness for the PURE PSU and PSU + 3% ZnO samples, revealing an intermediate roughness value for the PSU + 1% ZnO sample, with these values being given in Table 1. This result also tells us that surface roughness does not have much influence on wettability (since the sample with the highest surface roughness was the one without zinc oxide). Still, instead, the one with the best wettability was the one with the highest percentage of this material, as shown in Figure 3. In other words, wettability was increased by interaction with the zinc oxide particles added to the material, which, as mentioned earlier, has hydrophilic character. The increase in roughness, instead of improving wettability, decreases it. This contrary effect to the improvement of wettability is due to the accumulation of air in imperfections such as the pores formed in the samples (see Figure 5 a) with specific sizes, as explained by refs (38 and 39). This prevents liquids from penetrating such pores. In the case of the PSU + 3% ZnO sample, besides the better affinity with liquids due to the higher concentration of zinc oxide, the liquid absorbed in the upper layer can distribute into the pores inside this membrane. This explains why the best result was obtained in the contact angle test, as seen in Figure 3.

**Table 1:** Roughness Parameters

Roughness Parameters	Pure PSU	PSU + 1% ZnO	PSU + 3% ZnO
Ra (nm)	60.967	37.035	59.118
Rz (nm)	566.157	414.369	455.719
Rq (nm)	77.201	52.768	74.376
Rp (nm)	273.908	190.648	208.012

However, intermediate roughness is preferable due to its more homogeneous surface, which reduces obstructions and irregularities, resulting in better permeate flow than excessively rough or smooth membranes.^40,41^ Furthermore, this balanced roughness increases the effective surface area of the membrane, allowing for effective particle retention and optimized interaction with target molecules, making the separation or filtration process more efficient.^42^ It also consumes less energy due to the balance between retention and flow, resulting in significant energy savings in filtration and separation applications.^43^ The filtration test results corroborate this, as we will see in the effluent filtration analysis.

### 3.5 Effluent Filtration Analysis

In the filtration test, the highest amount of filtered liquid was obtained by the membrane with 1% ZnO, as can be seen in Table 2. This result indicates that wettability alone is not capable of improving this parameter. To better understand this result, analyzing the SEM results again is sufficient.

**Table 2:** Flux and Reduction of Effluent Color in the Permeate

Membrane	Flow (L/(m^2^·h))	Color Reduction (%)
Pure PSU	33.958 ± 1.132	82.89%
PSU + 1% ZnO	39.348 ± 1.083	83.72%
PSU + 3% ZnO	34.141 ± 2.112	82.73%

Checking the SEM micrographs, it is observed that the PSU + 1% ZnO membrane has two factors that tend to improve liquid permeability in the effluent filtration analysis, besides wettability, which, we recall, has an intermediate value among the three samples analyzed in this study. In this sample, both the upper and lower surfaces have much smaller thicknesses than do the others. Furthermore, the region between these two surfaces contains species of capillary channels interconnecting them. Another characteristic of these channels that may justify this better result is the capillary pressure effect, as explained in ref (36). These characteristics differ from the other two samples (PURE PSU and PSU + 3% ZnO) (see Figure 5).

This result showed an increase in the amount of filtered effluent of approximately 27.6%, indicating that the amount of ZnO that allows the formation of a membrane with ideal physical and chemical characteristics for filtration is 1% ZnO. However, this better filtration result is not reflected in a significant reduction in color, as can be seen in Table 2. The samples had practically equal values with a difference in color reduction percentage of only 0.83% between the sample without ZnO and with 1% of ZnO and the PSU + 3% ZnO samples, indicating that the addition of this oxide has little or no influence on this result.

**Figure 7:**
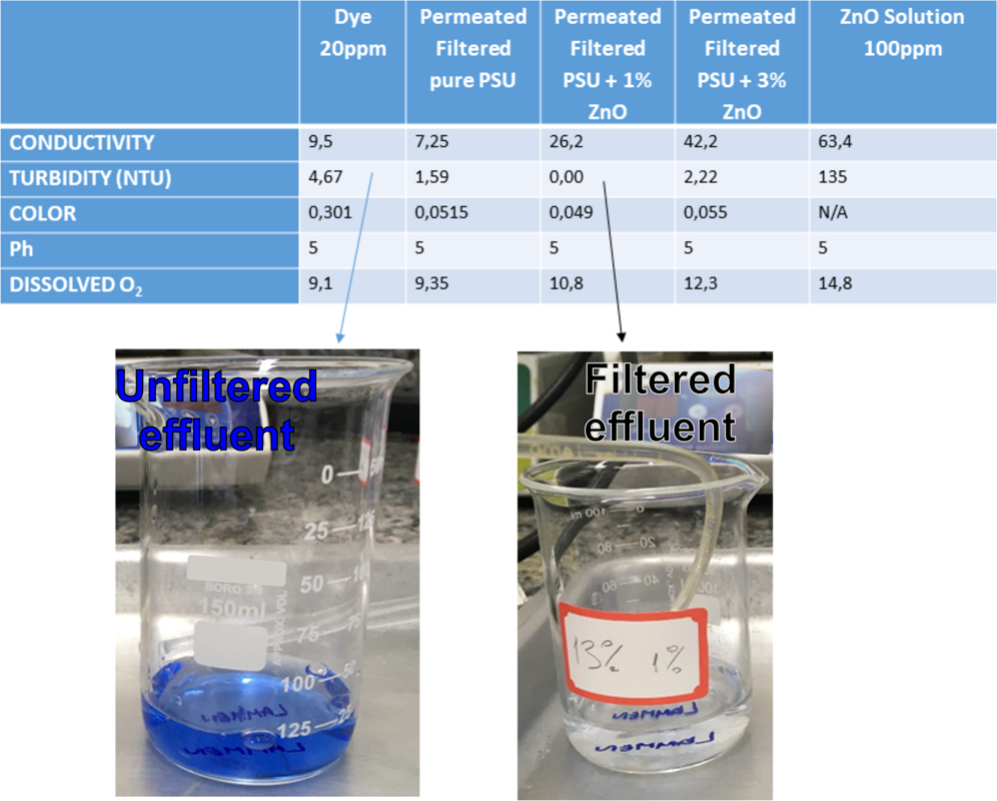
Characterizations of the dye, the solution with ZnO, and the permeated effluents.

Regarding the filtered effluents, Figure 7 presents the results of the analysis of the liquids before and after filtration on the membranes produced in this study. In the analysis of the conductivity data, it is noticed that the lowest value was obtained when filtration was performed by the membrane without the addition of ZnO, with successive increases proportional to the increase in the percentage of ZnO. This is because the oxide increases the electrical conductivity of the effluent due to the presence of the zinc metal. Still, the pH remains constant before and after the filtration process.

It can also be observed In Figure 7 that for the membrane with 1% ZnO, turbidity was reduced to zero. It is worth noting that these analyses were conducted in triplicate, and yet this result was consistent across all three measurements. This good result is followed by the filtrate of the pure membrane and, last, by the membrane with 3% ZnO. The increase in turbidity is also related to the increase in ZnO concentration since the ZnO solution exhibits a high degree of turbidity. The data confirming this can be found in Figure 7 (Top table), in the column with the analysis of the 100 ppm ZnO solution.

Additionally, the dissolved O2 coefficient of the permeates from all membranes increased after the filtration test. This is due to both the filtration process used and the addition of zinc oxide in the hybrid membranes. The filtration cell used in this study isolates the injected pressure to compress the present simulated effluent and accelerate the filtration step. Thus, all of the oxygen present in the cell is also incorporated into the permeated effluent during the filtration process, increasing its quantity of dissolved O2.

## 4 Conclusions

This study achieved satisfactory results regarding membrane filtration using polysulfone, demonstrating that zinc oxide has the potential to enhance this outcome, and showing that the addition of 1% ZnO yields optimal results in filtration. Another result indicated by this study is that wettability is not the only factor contributing to efficient liquid cleaning. The best wettability result was obtained with the addition of 3% ZnO, which did not translate into efficient cleaning of the solution, as seen in the effluent filtration result. This result showed that both lower turbidity and a higher volume of filtered solution were obtained with the membrane with an intermediate wettability value as well as apparent porosity, indicating that other factors may contribute to more efficient filtration. Thus, the parameters responsible for the effectiveness and efficiency of filtration include the perfect combination of wettability, roughness, and internal membrane structure, which in the case of this study was achieved with the membrane produced with 13% polysulfone and 1% zinc oxide.
